# A biochemical network can control formation of a synthetic material by sensing numerous specific stimuli

**DOI:** 10.1038/srep10274

**Published:** 2015-05-15

**Authors:** Ju Hun Yeon, Karen Y. T. Chan, Ting-Chia Wong, Kelvin Chan, Michael R. Sutherland, Rustem F. Ismagilov, Edward L. G. Pryzdial, Christian J. Kastrup

**Affiliations:** 1 Michael Smith Laboratories and Department of Biochemistry and Molecular Biology, University of British Columbia, Vancouver, BC, Canada; 2 Centre for Innovation, Canadian Blood Services, Vancouver, BC, Canada; 3 Centre for Blood Research and Department of Pathology and Laboratory Medicine, University of British Columbia, Vancouver, BC, Canada; 4 Division of Chemistry and Chemical Engineering, California Institute of Technology, Pasadena, CA, USA

## Abstract

Developing bio-compatible smart materials that assemble in response to environmental cues requires strategies that can discriminate multiple specific stimuli in a complex milieu. Synthetic materials have yet to achieve this level of sensitivity, which would emulate the highly evolved and tailored reaction networks of complex biological systems. Here we show that the output of a naturally occurring network can be replaced with a synthetic material. Exploiting the blood coagulation system as an exquisite biological sensor, the fibrin clot end-product was replaced with a synthetic material under the biological control of a precisely regulated cross-linking enzyme. The functions of the coagulation network remained intact when the material was incorporated. Clot-like polymerization was induced in indirect response to distinct small molecules, phospholipids, enzymes, cells, viruses, an inorganic solid, a polyphenol, a polysaccharide, and a membrane protein. This strategy demonstrates for the first time that an existing stimulus-responsive biological network can be used to control the formation of a synthetic material by diverse classes of physiological triggers.

Biological materials often form in a highly responsive manner, and organisms regulate these processes with precise control by integrating many environmental signals. This responsiveness is often the result of complex biochemical and genetic networks that sense numerous, diverse regulators^1^. These networks can amplify signals from appropriate stimuli with exquisite discretion. For example, the formation of blood clots is carefully controlled by a multitude of co-operative on- and off-switches. These switches regulate an intricate network of enzymatic reactions that polymerize and cross-link fibrin. Synthetic materials have not achieved this level of control^1^,^2^, partly because it is difficult to engineer numerous types of molecular recognition into the materials or their cross-linking systems. Current synthetic materials can typically only respond to one signal with high specificity^3^,^4^, or alternatively, many stimuli but with the inability to distinguish between similar signals^5^,^6^,^7^. For example, there are synthetic materials that form directly in response to a specific purified cross-linking enzyme^8^, but their formation lacks sensitivity to the plethora of stimuli that naturally controls the enzyme in its biological system. For this reason, there is tremendous interest in creating “smart” materials that are responsive to multiple signals, with a goal toward developing materials that can respond to environmental cues^2^. Many successful strategies have been developed^1^,^9^,^10^, but smart materials do not typically detect distinct stimuli from more than three to four diverse classes of signals^2^. It can be particularly challenging to control the formation of a material through polymerization or self-assembly by multiple diverse stimuli, while the behaviour of the material, such as swelling, degradation or localization, can more often be controlled^5^,^6^,^7^. We are unaware of a material whose formation is responsive to specific stimuli from over eight diverse classes of signals.

We asked the question: Can the responsiveness of a material be expanded if its cross-linking enzyme is connected to a biological network? To test this in proof-of-concept experiments for this strategy we used components that were previously well-characterized^11^,^12^,^13^,^14^,^15^. We used a synthetic material, a polyethylene glycol (PEG) hydrogel, whose polymerization is catalyzed by coagulation factor XIIIa (FXIIIa)^11^,^12^,^13^,^14^,^15^. FXIIIa is a transglutaminase that covalently cross-links glutamine to lysine residues or to other primary amines, and is a promiscuous enzyme with many known substrates. PEG macromers conjugated to appropriate peptides can be cross-linked by purified FXIIIa in buffered systems to form gels^11^,^12^,^13^,^14^,^15^. FXIIIa plays an important role in blood coagulation. During the coagulation process, the zymogen factor XIII (FXIII) is cleaved by the protease, thrombin, and activated to form FXIIIa, which then cross-links and stabilizes fibrin, the natural output of the coagulation system. In blood plasma, the activity of thrombin is controlled by a network of dozens of enzymatic reactions, which constitute a sensor that indirectly controls the activation of FXIII and cross-linking of fibrin^16^. We tested if this ability of the biological network to indirectly control cross-linking of fibrin could be used to control cross-linking of the synthetic material, by mixing the synthetic macromer with fibrinogen-depleted plasma. By connecting these well-characterized components, we hypothesized that the biological network would allow the synthetic material to indirectly polymerize in a clot-like manner in response to new stimuli.

We found that this PEG hydrogel was formed as the end product of the coagulation cascade, in place of fibrin. Although the blood coagulation network is composed of dozens of reactions that could have potentially been impeded by replacing fibrinogen with high concentrations of a synthetic macromer, the network remained functional. The network retained the ability to sense the vast and specific repertoire of natural regulators of coagulation to robustly form and degrade this material. The material was then controlled not just by the direct addition of FXIIIa, or FXIII and thrombin, but also indirectly by the multitude of diverse stimuli that can modulate the coagulation network through thrombin and FXIIIa. The “biochemical network connected to the formation of a material” (BNC) polymerized the synthetic material in response to all of the system’s natural input triggers we evaluated, including specific chemicals, materials, cells, and combinations of stimuli. This BNC-material exhibited mechanical properties that were distinctly different from the biological network’s natural output of fibrin, such as greater stiffness and structural stability. In this strategy, the responsiveness of the modified system emerged nearly entirely from the biological network and this responsiveness was passed on to the cross-linking enzyme and then indirectly to the material, leading to its clot-like polymerization. This approach of changing the output of a biological network resembles strategies used in synthetic biology, where networks in microorganisms are altered to produce new biological products or gain new functions^17^,^18^,^19^. Although the approach and the results are in some ways rather intuitive and expected, the outcome is remarkable in that it is the first liquid mixture we are aware of that responds to specific modulators from over eight diverse classes of signals to harden into a synthetic material.

## Results

**Figure 1:**
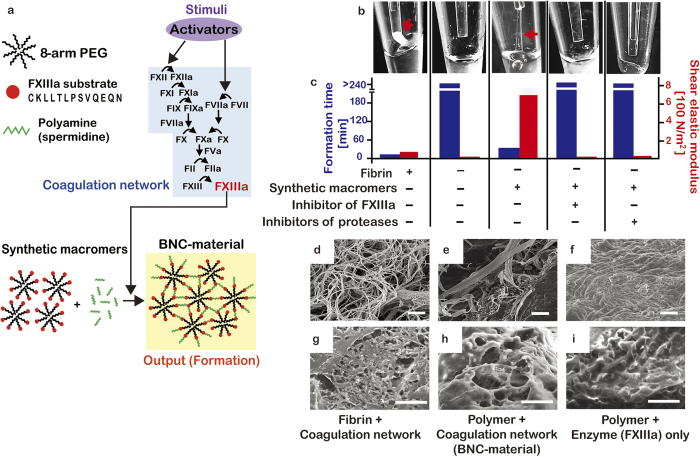
The biochemical reaction network of blood coagulation is a sensor that can be used to polymerize a synthetic material in a clot-like manner. (**a**) Schematic showing an input of activators (purple) into the coagulation enzyme network (blue) generating the cross-linker, FXIIIa, and a clot-like BNC-material (yellow) upon addition of the synthetic macromer. (**b**,**c**) Clot-like polymerization of liquid mixtures containing the coagulation network and either fibrin or the synthetic macromers. Images of microtubes (**b**) show clot-like gels adhered to pipette tips and pulled upwards when fibrin or the synthetic macromer was connected to the network. Gels did not form when inhibitors of coagulation enzymes were present. The gel formation times (blue bars) and shear elastic modulus (red bars) are shown in (**c**) Formation times are mean ± SEM with *n* = 3-5. (**d**–**i**) Scanning electron micrographs (top row) and cryo-scanning electron micrographs (bottom row), of fibrin in normal plasma (**d**,**g**), the BNC-material (**e**,**h**) and macromer polymerized with FXIIIa but without the network (**f**,**i**) Scale bars represent 20 μm.

To test whether the coagulation network could exert control over the formation of a synthetic material that can be polymerized by FXIIIa, the PEG macromer and a polyamine were added to human plasma lacking fibrinogen, the soluble precursor of fibrin (Fig. 1a). This fibrinogen-deficient plasma enabled us to selectively detect gelling of the synthetic macromer, rather than fibrin. Purified FXIII zymogen was added due to co-depletion of FXIII along with fibrin. The synthetic macromer was constructed as described by others, by conjugating an 8-armed PEG to a peptide sequence found in α_2_-antiplasmin, a natural, glutamine-donating, plasma protein substrate for FXIIIa^20^. Spermidine, a polyamine, was added with the synthetic macromer to fibrinogen-depleted plasma, serving as the amine-donating substrate for FXIIIa. Other polyamines could be substituted for spermidine (Supplementary Fig. S1). When blood coagulation was triggered with silica to activate the factor XII branch of the coagulation network, FXIIIa cross-linked the amine- and glutamine-donating substrates (spermidine and the α_2_-antiplasmin peptides, respectively) polymerizing the BNC-material in a clot-like manner. The shear elastic modulus increased over 40-fold during formation. The BNC-material also displayed mechanical properties that differed from fibrin. Fibrin has a low elastic modulus and exhibits clot retraction, contracting to a fraction of its size when physically disturbed, which limits its use as a material for some applications. The shear elastic modulus of the BNC-material was over 10-fold higher than a fibrin clot (Fig. 1b,c). Furthermore, the BNC-material was more stable than fibrin, as it did not retract or permanently deform when handled. Polymer formation in fibrinogen-deficient plasma occurred only when both the macromer and the amine-donating spermidine were present. An inhibitor of FXIIIa or a cocktail of serine protease inhibitors prevented polymer formation, verifying that formation was indirectly controlled by the coagulation system. Using both conventional scanning electron microscopy (SEM) and cryo-SEM, the architecture of the BNC-material was compared to that of the synthetic polymer that was cross-linked with purified FXIIIa in the absence of plasma and other coagulation enzymes (Fig. 1d–i). The BNC-material better resembled the fibrous and porous structure of fibrin. This fibrous structure may have emerged from plasma proteins providing a template as the synthetic polymer formed, or from the spatial-heterogeneity of enzyme activation that occurs during coagulation^21^,^22^,^23^,^24^.

**Figure 2:**
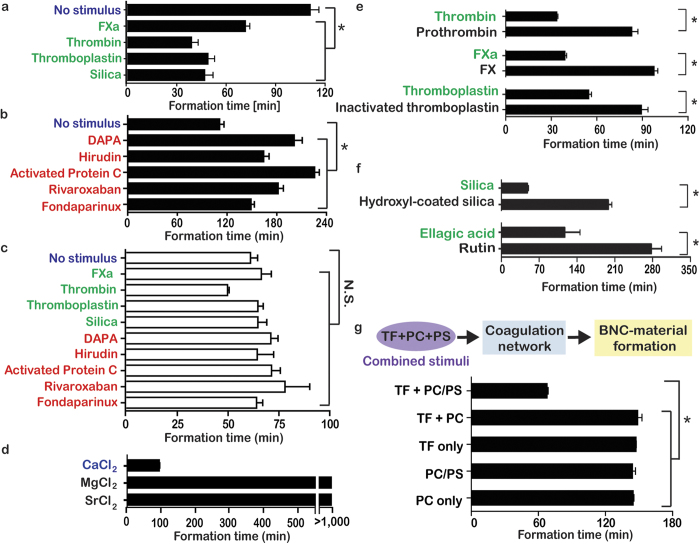
Clot-like polymerization of the BNC-material can be controlled by diverse and specific modulators when coupled to a network. (**a**,**b**) The formation times of the BNC-material in indirect response to known activators (**a**) or inhibitors (**b**) of coagulation, compared to the control without a stimulus. (**c**) The formation times without the network, using purified FXIIIa, did not respond to the modulators (all p > 0.05). (**d–f**) The formation times by activators compared to agents that have similar chemical structures, indicating that the formation of BNC-material was specific to its modulators. The formation of the BNC-material was selective to calcium ions (**d**) the active forms of coagulation enzymes (**e**) and contact pathway activators (**f**). (**g**) Schematic and graph showing that formation was indirectly sensitive to combinations of stimuli, and BNC-material formation was fastest when TF, PS and PC were combined together. All data indicate mean ± SEM, n = 3-5, *p < 0.05.

Coagulation is responsive to several distinct classes of stimuli, including small organic molecules, divalent metal ions, the extracellular matrix, anionic lipid surfaces, and soluble and transmembrane proteins. To test if the formation of the material indirectly responds to diverse stimuli when coupled to the coagulation network, its formation time was measured after adding known activators and inhibitors of coagulation. The activators included two enzymes, coagulation factor Xa and thrombin, thromboplastin (a mixture of transmembrane tissue factor (TF) embedded in phosphatidylserine (PS)/phosphatidylcholine (PC)-containing vesicles with CaCl_2_), and inorganic silica nanoparticles. The inhibitors included two small molecules, rivaroxaban and dansylarginine N-(3-ethyl-1,5-pentanediyl)amide HCl (DAPA), a small protein, hirudin, an enzyme, activated protein C, and a polysaccharide, fondaparinux. DAPA and hirudin bind and inhibit thrombin, rivaroxaban binds and inhibits FXa, APC is a proteolytic inhibitor of coagulation cofactors, and fondaparinux accelerates coagulation enzyme inhibition. These 9 diverse and specific modulators of coagulation significantly altered the formation time of the BNC-material. Clot-like polymerization occurred in 120 min in the absence of a stimulus, but occurred approximately twice as fast with an activator and significantly slower with an inhibitor (Fig. 2a,b). These same activators and inhibitors respectively sped up and slowed down fibrin clot formation in normal plasma (Supplementary Fig. S2). Although they influenced formation times, these modulators did not significantly affect the final material’s physical properties, such as the compressive elastic moduli (Supplementary Fig. S3). When the macromer was not coupled to the network, but was polymerized with purified FXIIIa alone, the stimuli did not cause significant differences in formation time (Fig. 2c). Thus, the original FXIIIa-cross-linkable material that was described previously could be made responsive to numerous new stimuli by connecting it to the coagulation network. The sensitivity of the BNC-material to activators could be modulated by changing the concentrations of coagulation factors (see supplementary methods and Table S1). By increasing the concentration of coagulation enzymes, another activator, ellagic acid, a plant polyphenol, could be detected by the BNC-material. The clot-like polymerization of the BNC-material was highly specific to its modulators and could distinguish between closely related signals (Fig. 2d–f). Prothrombin and factor X (the respective zymogens of thrombin and FXa), heat-inactivated thromboplastin, silica nanoparticles with hydroxlyated surfaces, and rutin (a plant polyphenol) have similar chemical structures to their counterparts, but led to significantly slower formation of the BNC-material. The enzymes of the coagulation system also specifically require calcium ions as opposed to other divalent cations, as formation of the material did not occur when calcium was replaced with magnesium or strontium.

The coagulation network can sense and respond to combinations of stimuli, which allow clotting to respond appropriately at sites of vascular damage without causing errant clotting and thrombosis. Sensing and responding to specific combinations of stimuli is challenging to engineer in synthetic materials. To evaluate if the network can be used to extend the repertoire of signals recognized by the original FXIIIa-cross-linkable material to include specific combinations of stimuli, the functional contribution of thromboplastin constituents were dissected. Formation of the BNC-material was sensitive to combinations of TF, PS, and PC, and only occurred when all three were combined (Fig. 2g), which is consistent with the sensitivity of the endogenous coagulation system.

**Figure 3:**
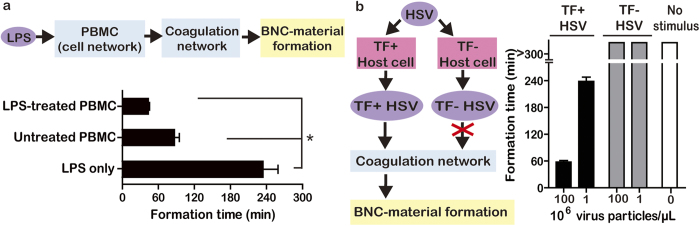
Cells and viruses with specific phenotypes can stimulate the BNC-material to polymerize in a clot-like manner. (**a**) The BNC-material formed faster when cells were stimulated with lipopolysaccharide (LPS), indicating sensitivity to the activation state of PBMC, *p < 0.05. (**b**) The formation of the BNC-material could indirectly distinguish between two variants of herpes simplex virus (HSV), and formed faster when HSV containing TF on its surface was added as a stimulant compared to HSV that did not express TF. All data indicate mean ± SEM, n = 3.

Materials in nature can respond to subtle differences in the phenotypes of cells and viruses in their environments. We tested if formation of the BNC-material could distinguish between activated and non-activated peripheral blood mononuclear cells (PBMC). PBMC activate when treated with lipopolysaccharide (LPS), a common bacterial antigen that induces the expression of TF. Formation of the BNC-material occurred faster in the presence of activated PBMC compared to resting PBMC that were not pre-stimulated by LPS (Fig. 3a). Formation in the presence of resting PBMC also occurred faster than without PBMC, but not as fast as with activated PBMC.

To further examine if the BNC-material retained the ability to detect minute changes in biological stimuli, two variants of herpes simplex virus (HSV) were compared, one with and one without host-cell derived TF incorporated in the envelope surface. HSV is a known stimulus of clotting if the host cell that replicates the virus expresses TF^25^. To produce the two variants of HSV, host cells that were otherwise identical were engineered to either express TF (TF+) or not express TF (TF-) and then were inoculated with HSV. The BNC-material detected this variation between the HSV and formed faster in the presence of the TF+ variant (Fig. 3b). It is remarkable that formation could detect the small difference between these two viruses, whose genotypes were identical but which were grown in host cells with or without a single gene knockout.

**Figure 4:**
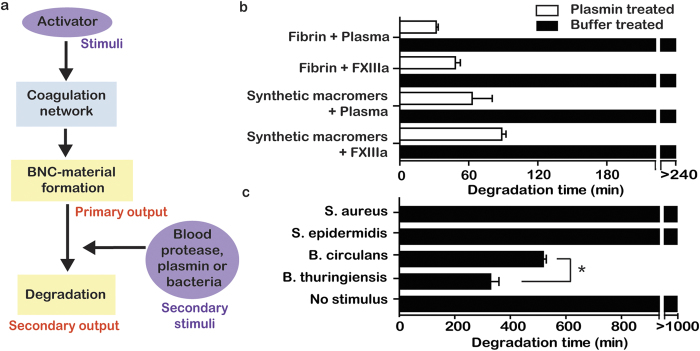
The BNC-material was controllably degraded by specific modulators. (**a**) Schematic showing that the addition of a blood protease, plasmin, or bacteria degrades the BNC-material. (**b**) Plasmin degrades the BNC-material with or without fibrin present. (**c**) The material degraded in the presence of *Bacilli* strains, but not in the presence of *Staphylococci* strains. Data indicate mean ± SEM, n = 3, ^*^P < 0.05.

In contrast to solidification, there are many examples of synthetic materials that can be degraded in response to particular cells^26^,^27^. Peptide-containing materials, such as the synthetic macromer used in this report, are sensitive to degradation by proteases. In blood, fibrin is naturally degraded by the serine protease plasmin and bacterial proteases^28^, which can both degrade a wide range of peptides. We tested if the preformed BNC-material could also be degraded by plasmin or bacteria (Fig. 4a). The BNC-material was degraded by exogenous plasmin within 100 min, which was similar to fibrin gels formed under the same conditions (Fig. 4b). The synthetic gels formed in the absence of plasma degraded at a similar rate to those that contained plasma, suggesting that cleavage occurred at the synthetic peptides. Degradation of the BNC-material was sensitive to bacterial strains. Degradation was not apparent in the presence of two *Staphylococcus* strains, but did occur by, and could distinguish between, two strains of *Bacilli* (Fig. 4c). This could be due to differences in the type or concentration of proteases produced by these strains. In the future, altering the peptide sequences in the BNC-material presents potential for tuning the material’s mechanical and degradation properties for specific applications.

## Discussion

Here, we demonstrate for the first time that a biological network can be used to control the formation of a synthetic material, allowing the material to then indirectly respond to specific stimuli from more than 8 diverse chemical and biological classes. Formation indirectly responded to even subtle changes in the overall phenotype of a virus. These proof-of concept experiments utilized well-known and well-characterized components; the biological network and sensor was the blood coagulation cascade from plasma, and the synthetic material was a FXIIIa-cross-linkable PEG macromer. Although with or without the biological sensor the materials were very similar after they were formed, the clot-like polymerization of the material was only modulated by diverse stimuli when the biological sensor was present. It is remarkable that the controllability over a synthetic polymer could reach a level of precision typically only seen in nature simply by mixing these well-characterized components together^1^,^2^. This approach mimics strategies used in synthetic biology, where microorganisms have been engineered to produce new molecules from their existing biological networks or to produce their metabolites in response to new stimuli^17^,^18^,^19^. The simple approach to extending the responsiveness of existing materials described here is different from approaches that chemically modify their backbones^1^. In fact, the responsiveness of the synthetic material was increased without requiring drastic changes to the chemical composition of the final polymer. In the approach here, the material does not directly respond to multiple stimuli, but responds indirectly through a network and sensor that modulates its cross-linking agent. Specifically, modulators of the coagulation cascade primarily influence the rate of formation of FXIIIa rather than its catalytic efficiency^29^. It is expected that modifying the substrates and the catalytic activity^30^ would influence the magnitude of the formation times, but not the selectivity to the modulators. Modifying the polymer backbone, such as changing the length or branching of PEG, is expected to influence the physical properties of the final material^31^, but not the sensitivity of the system to the coagulation modulators. Strategically changing the polymer backbone to add recognition and sensitivity to other modulators, is a potential strategy for further increasing the responsiveness of the system^13^. Thus, smart materials based on polymers with responsive backbones could be complementary to the approach herein. The strategy described here is also distinguishable from, and potentially complementary to, approaches used to create functional materials through biomimicry. Biomimicry, imitating the dynamics and functions of biological materials, is a proven strategy to engineer new and useful materials^32^,^33^. Together these approaches may be capable of creating multi-functional smart materials that emulate the highly evolved and tailored reaction networks of complex biological processes.

With further development, this specific BNC-material may have applications in medicine, arising from its ability to mimic aspects of fibrin/ogen and form in a clot-like manner in response to modulators of coagulation. Blood clotting is a tightly-regulated process—normally activating only where and when it is supposed to. Retaining this regulation while replacing the output from a fibrin clot to a synthetic material may be useful for developing smart tissue-adhesives. It may be particularly useful as an intravascular drug depot, where it could potentially be coated onto local areas of the vasculature with a catheter, as has been done with other gels in order to heal atherosclerotic plaques^34^. Alternatively, it may be useful in other applications. This tight biological regulation may allow it to be developed into a tool for detecting signals from specific cellular, viral, or bacterial pathogens that modulate clotting. Also, an appropriate synthetic equivalent of fibrin/ogen, developed based on the BNC-material strategy, would be useful for treating fibrinogen deficiency by replacing fibrin’s role in coagulating at vascular damage. Fibrinogen deficiency can occur congenitally and acutely from trauma or sepsis, resulting in severe bleeding episodes^35^.

Although there are a number of potential applications, questions remain regarding the feasibility and extensibility of the BNC strategy. In theory, any biochemical cascade resulting in the output of a cross-linking enzyme, including other transglutaminases, can be exploited to exert biological control over the polymerization of synthetic materials bearing substrates for the enzyme. Furthermore, this strategy can possibly be extended to biochemical cascades that do not produce cross-linking enzymes, such as those that produce proteases capable of activating the self-assembly of materials. A limitation of these strategies is that biological networks typically function under tightly controlled conditions, which may limit their use to specific biological settings. For example, although the coagulation network can function outside of blood vessels, applications of the BNC-material investigated here may be limited to the inside of blood vessels or to highly vascularized tissues. Identifying other biological networks that can modulate the activity of cross-linking agents or self-assembling processes^36^, and developing strategies that allow them to function outside their native environments is a potential route to reformulating useful synthetic materials to attain powerful new functions.

## Methods

### 

See supplementary information for a detailed description of the materials and methods

#### Synthesizing and forming the BNC-material

A peptide sequence derived from α_2_-antiplasmin, acetyl-NQEQVSPLTLLKKGC, was conjugated through its cysteine thiol to malemide groups on eight arms of branched PEG (Supplementary Fig. S4). In a buffered solution (10 mM HEPES, 7 mM sodium citrate, pH 7.4), the macromer (87 mg/mL) was polymerized in a reaction mixture containing human FXIIIa (3.7 μM), spermidine (2.4 mM), DTT (0.90 mM), and CaCl_2_ (9.4 mM) (Supplementary Fig. S5). When the BCN-material was formed in plasma, the reaction mixture contained synthetic macromer (49 mg/mL), fibrinogen-deficient plasma (13.3% v/v), purified FXIII (0.29 μM), spermidine (1.8 mM), DTT (0.66 mM), and CaCl_2_ (29 mM) in HEPES buffer. Plasmas deficient in fibrinogen and other factors were obtained from Affinity Biologicals. The reaction mixtures were incubated at 37 C with various concentrations of stimuli (Supplementary Table S2).

#### Measuring BNC-material formation

Thromboelastography (TEG) was used to determine the shear elastic moduli of the materials during formation (Supplementary Fig. S6). The BNC-material reaction mixture or normal plasma was recalcified and 300 μL was monitored for 3 hr at 37 °C by TEG. The materials’ compressive elastic moduli were measured after formation by a controlled-force compression test (Supplementary Fig. S3). The BNC-material (5.79 μL) was formed overnight at 37 °C, and its stress and strain were measured using a TA Q800 dynamic mechanical analyzer. The formation times in response to stimuli were measured in microwells using fluorescent beads added into the mixture (6 × 10^8^ particles/mL). The Brownian motion of fluorescent beads was monitored using an epi-fluorescence microscope, and cessation of movement indicated polymer formation. The microchambers were constructed using an adhesive silicone isolator sandwiched between two plastic coverslips, and each well held 5 μL of the reaction mixture or plasma (Supplementary Fig. S7). Degradation of the BNC-material or other preformed gels was measured by adding plasmin (333 μg/mL) (Supplementary Table S3) or bacteria (36 μg/mL) to samples formed in microchambers and measuring the time required to liberate trapped fluorescent beads. All *p* values were calculated by two-tailed Student’s-*t* test.

#### Isolation, Culture, and FACS Analysis of PBMC

PBMC were collected from human whole blood by a lymphocyte isolation density gradient and resuspended in culture medium (RPMI 1640; 50,000 U/L penicillin; 50 mg/L streptomycin; 25 mM HEPES; 20% FBS) at 10^6^ cells/mL. Lipopolysaccharide (LPS) was added to cells at 1 μg/mL. The cells were incubated at 5% CO_2_ and 37 ˚C for 24 hr. Cells +/- LPS treatment were added to the BNC-material reaction mixture as a stimulus at a final concentration of 1.3 × 10^5^ cells/μL. Remaining cells were stained with anti-Human CD142 PE and propidium iodide for FACS analysis (Supplementary Fig. S8).

#### Preparing TF+ HSV-1

A low-passage clinical isolate, HSV-1 NS, was propagated in human melanoma cell line A7 that either expresses or is deficient in TF. Viral progeny were isolated by sucrose gradient ultracentrifugation^25^ and added to the BNC-material reaction mixture as an initiator.

## Author Contributions

J.H.Y., K.Y.T.C., T.C.W., R.F.I., E.L.G.P., and C.J.K. conceived the hypotheses, methods, and applications; K.Y.T.C., J.H.Y., T.C.W., K.C., M.R.S., and C.J.K. performed experiments; K.Y.T.C., J.H.Y., T.C.W., K.C., and C.J.K. analyzed data; K.Y.T.C., J.H.Y., T.C.W., and C.J.K. wrote the manuscript; and all authors discussed results and commented on the manuscript. J.H.Y. and K.Y.T.C. contributed equally.

## Additional Information

**How to cite this article**: Yeon, J. H. *et al*. A biochemical network can control formation of a synthetic material by sensing numerous specific stimuli. *Sci. Rep.* **5**, 10274; doi: 10.1038/srep10274 (2015).

## Supplementary Material

Supplementary Information
