# Supplementary material for: A biochemical network can control formation of a synthetic material by sensing numerous specific stimuli
Source: Sci Rep. 2015 May 15;5:10274. doi: 10.1038/srep10274 (PMC4432564; doi:10.1038/srep10274)
Supplement: Supplementary Information [file srep10274-s1.pdf]

SUPPLEMENTARY INFORMATION FOR

A biochemical network can control formation of a synthetic material by sensing numerous specific stimuli

Ju Hun Yeon[†], Karen Y. T. Chan[†], Ting-Chia Wong, Kelvin Chan, Michael R. Sutherland, Rustem F. Ismagilov, Edward L. G. Pryzdial, Christian J. Kastrup

Materials and Methods

Synthesizing the synthetic macromer.

The acetyl-NQEQVSPLTLLKKGC (Thermo Fisher Scientific, Waltham, USA) peptide derived from α2-antiplasmin was conjugated to polyethylene glycol maleimide (PEG-mal) (40 kDa, 8-armed) (Creative PEGWorks, Winston Salem, USA). To reduce disulfide coupling of the peptides, the peptide solution (20 mg/mL, 12.5 mM) was run through an immobilized Tris-(2-carboxyethyl)phosphine (TCEP) disulfide reducing gel (Thermo Fisher Scientific). For the conjugation reaction, the peptides were reacted with PEG-mal (50 mg/mL, 1.25 mM) in Tris-EDTA buffer (150 mM NaCl, 50 mM Tris-Base, 10 mM EDTA, pH 8) overnight at 4^{0}C, stirring continuously. To quench any remaining unreacted maleimide, mercaptoethanol (180 µM) was added to the mixture. This mixture was then dialyzed against Tris-EDTA buffer for 1 day, and then against water for 2 days. To confirm the conjugation of PEG-mal and peptides, Ellman's reagent (5,5'-dithiobis(2-nitrobenzoate) (DTNB), Sigma), a sensitive reagent for measuring free sulfhydryl content, was used to detect free thiols on the peptide. Free thiols on the peptide significantly decreased and were eliminated following conjugation with PEG-mal and subsequent dialysis. The dialyzed solution was purified further using a desalting column (Zeba Spin Desalting Columns, 7k MWCO, Thermo Fisher Scientific) and then the purified conjugates were concentrated by a Centri-prep filter (Amicon®Ultra 10K, Millipore). The final conjugates were lyophilized and stored at -20^{0}C until use. The molecular weight of the modified PEG-mal was measured by Matrix-Assisted Laser Desorption Ionization-Tandem Time of Flight (MALDI-TOF) to confirm conjugation with peptides, comparing it to the molecular weight of unmodified PEG-mal (see **Supplementary Fig. S4**). The size of the mass shift indicated that approximately all the maleimide groups conjugated to peptides.

Testing the rate of fibrin clot formation in response to known modulators.

Frozen normal human plasma was thawed at 37^{0}C and recalcified. The recalcification solution (90 mM NaCl and 40 mM $CaCl_2$) was added to the plasma in a 1:3 ratio. Each modulator was separately added to this mixture (concentrations stated in **Supplementary Table S2**) and samples were maintained at 37^{0}C.

Testing the formation time of the BNC-material in response to modulators.

A reaction mix was prepared in buffer (10 mM HEPES, 7 mM sodium citrate, pH 7.4) containing the synthetic macromers (49 mg/mL), fibrinogen-deficient plasma (13.3% v/v)

(Affinity Biologicals, Ancaster, Canada), purified FXIII (93 μg/mL) (Haematologic Technologies Inc., Vermont, USA), spermidine (1.8 mM) (Sigma-Aldrich, St. Louis, USA), dithiothreitol (DTT; 0.66 mM) (Sigma-Aldrich, St. Louis, USA), and $CaCl_2$ (29 mM) (Acros Organics, New Jersey, USA). The stimuli being evaluated were added to this mixture at the test concentrations stated in **Supplementary Table S2** and the reaction was maintained at 37^0C. Fibrinogen-deficient plasma partially depleted for other coagulation factors was used in **Fig. 2d, e, g** and **3**. Specific enzyme activities for this plasma are listed in **Supplementary Table S1**. A higher concentration of fibrinogen-deficient plasma (26.6% v/v) and lower concentration of supplemented purified FXIII (46.5 μg/mL) was used in **Fig. 2f**. The gelation time was consistent over a wide range of concentrations for most stimuli (**Supplementary Table S2**).

Gelling the synthetic macromer using purified FXIIIa, without plasma.
The same protocol for forming the BNC-material was used with minor modifications. The reaction mixture was made in the same citrated buffer containing the synthetic macromers (87 mg/mL), human FXIIIa (0.31 mg/mL, unless otherwise stated) (Haematologic Technologies Inc., Vermont, USA), spermidine (2.4 mM), DTT (0.90 mM), and $CaCl_2$ (9.4 mM). No blood plasma was added. In the experiments in which different stimuli were tested, stimuli were added to this mixture at concentrations specified in **Supplementary Table S2**. The reaction mixture was maintained at 37^0C.

Assessing the mechanical properties of the synthetic polymer and fibrin clots.
The mechanical properties of the BNC-material and fibrin clots were measured during the course of their formation by thromboelastography (TEG) (TEG® 5000 Thrombelastograph® Hemostasis System, Haemoscope Corporation, IL) (see **Fig. 1** and **Supplementary Fig. S6**)[1]. TEG measures properties such as the reaction rate, mechanical strength, and stability of gel or clot by measuring rotational movement of a pin suspended in a gel mixture or plasma. Reaction time (R) indicates the time that gelation begins. Amplitude (A) is a function of clot strength or elasticity. Maximum amplitude (MA) represents the maximum strength of the gel. The amplitude can be used to determine the shear elastic modulus (G) of gel or fibrin clot during their formation. The reaction mixture (300 μL) of the BNC-material or normal plasma was prepared as previously described and the reaction was initiated by recalcification and the addition of silica (Kontact reagent) to activate the coagulation network. The effects of an inhibitor of FXIIIa (10 mM 1,3,4,5-Tetramethyl-2-[(2-oxopropyl)thio]imidazolium chloride (T101), ZEDIRA GmbH, Germany) or a cocktail of protease inhibitors (2 mM 4-(2-aminoethyl)benzenesulfonyl fluoride, 0.3 μM aprotinin, 130 μM bestatin, 1 mM EDTA, 14 μM E-64, 1 μM Leupeptin, Sigma-Aldrich) were evaluated. The samples were analyzed at 37°C for 3 hr.

The compressive elastic moduli of the BNC-material gels were measured by a controlled force compression test (DMAQ800, TA Instruments, DE). The same protocol for forming the BNC-material was used as described above, with individual reaction volumes of 5.79 μL. The BNC-

material reaction mixtures were incubated overnight at 37°C to allow them to completely gel. Each gel was removed from its microchamber and placed in a submersion-compression clamp. Its diameter was measured using a caliper to determine the surface area of the sample. Preload force of 0.01 N was applied to the gel to ensure good contact with the compression clamp. The instrument measured initial sample thickness, ramped the compression force at 0.01 N/min and measured the sample's stress and strain. The compressive elastic modulus was calculated by taking the absolute value of the slope of the stress-strain curve at its initial linear portion where elastic deformation was observed (**Supplementary Fig. S3**).

Assessing the gelation of the BNC-material and normal plasma clots.

The BNC-material or normal plasma clots were formed in polypropylene tubes. The gelation time was determined to be the time it took for the reaction mixture to reach a viscosity at which it could no longer be pipetted (see photos in **Fig. 1b**). For measuring the gelation time of many samples simultaneously, a microscope assay was employed. Here, fluorescent beads (Fluoresbrite Plain YG 1.0 Micron Microsphere, Polysciences Inc., Warrington, USA) were suspended in the reaction mixture (6×10^8 particles/mL) and then transferred into individual microchambers. These microchambers were constructed by sandwiching an adhesive silicone isolator (4.5 mm diameter x 0.5 mm depth; Grace Bio-Labs, Bend, USA) between two plastic coverslips (HybriSlip, Grace Bio-Labs, Bend, USA) (**Supplementary Fig. S7**). Brownian motion of the green fluorescent beads within the reaction mixture was monitored via time-course imaging using a Leica DMI6000B fluorescence microscope. Gelation was considered to be complete when the fluorescent beads stopped moving.

Imaging the BNC-material and fibrin clots by electron microscopy.

Samples for conventional SEM were prepared as described previously[2]. The gels were fixed with formalin and then dehydrated with ethanol. These gels were carbon-coated and then imaged with a Hitachi S-4700 field Emission Scanning Electron Microscope. SEM imaging was replicated three times. Cryo-EM was done following previously published methods with slight modification[3]. The gels were flash-frozen in liquid nitrogen and excess water from the samples was sublimed in a higher temperature vacuum chamber. The samples were then carbon-coated prior to imaging.

Generating lipid vesicles.

Two lipids were used for the vesicles: porcine brain phosphatidylserine (PS) (Avanti Polar Lipids, Alabaster, USA) and 1,2-dilauroyl-sn-glycero-3-phosphocholine (PC) (Avanti Polar Lipids, Alabaster, USA). Two types of vesicles were made using these lipids, vesicles containing 100% PC, or negatively-charged PC/PS (molar ratio 4:1) vesicles. To make these, the lipids were dissolved in chloroform within 1.5 mL polypropylene tubes. The chloroform solution was spread evenly over the tube surface, and chloroform was evaporated off the tube surface using a stream of N_2 gas. Residual chloroform was evaporated overnight in a vacuum

chamber. The lipids were then resuspended in deionized H_2O (5 mg/mL for chemical stimuli assays, or 10 mg/mL for coating in microfluidic devices). To obtain vesicles of a specific diameter (100 nm), samples were passed through membranes of successively smaller pore sizes (Northern Lipids, Burnaby, Canada) three times using a Lipex Thermobarrel Extruder (Northern Lipids, Burnaby, Canada). The vesicles extruded off the final 100 nm membrane were used for the experiments.

Isolation, Culture, and FACS Analysis of PBMC.

This study was approved by the research ethics boards of the University of British Columbia, and informed consent was obtained from all healthy volunteers before whole blood donation. Whole blood was collected into tubes containing sodium citrate (12 mM) before 1:1 dilution in buffer (PBS with 2% FBS). The mix was layered onto lymphocyte isolation density media (Ficoll-Paque PLUS, GE Healthcare Bio-Sciences AB, Uppsala, Sweden) and the PBMC were isolated according to previous literature with minor modifications[4]. The layered blood was centrifuged at 400 x g for 30 min at room temperature. The PBMC layer was collected. Two washes at 200 x g were performed in a buffered solution (PBS with 2% FBS) to remove the platelets from the supernatant. The PBMC pellet was then resuspended in culture medium (RPMI 1640; 50,000 U/L penicillin; 50 mg/L streptomycin; 25 mM HEPES; 20% FBS) at 1 x 10^6 cells/mL. Cultures of 5 mL were either PBS-treated as an unstimulated control or stimulated with 1 µg/mL LPS. The cells were then incubated at 5% CO_2 and 37˚C for 24 h. For each sample, 10^6 cells were spun down and resuspended in 1 µL of HEPES-citrate buffer. The cells were then added to the coagulation-controlled gel reaction mixture. To confirm expression of TF remaining cells were stained with Anti-Human CD142 PE (eBioscience, San Diego, CA) fluorescent antibody on ice for 30 min. Propidium iodide was also added just before FACS analysis to stain for inviable cells. The cells were analyzed using FACS Calibur (BD Biosciences, San Jose, CA). Based on the light scatter, which indicates the cell size and granularity, PBMC were selected for analysis. Single color controls were used to determine the background fluorescence level of each stain. Based on this information, quadrants were drawn to indicate which cells were fluorescing above the threshold. Target cells, which were both viable and expressed TF, were shown in the bottom right quadrant (**Supplementary Fig. S8**).

Measuring plasmin-induced and bacteria-induced degradation of the BNC-material.

Human plasmin (Haematologic Technologies Inc., Vermont, USA) was diluted in HEPES buffer and added to 3-µL BNC-material or fibrin clots (final plasmin concentration 333 µg/mL) pre-formed in microchambers. Microchambers were then sealed with immersion oil (type DF, code 1261) (Cargille Laboratories, Inc., Cedar Grove, NJ) and the gels were monitored for lysis at 37°C. Degradation time was determined by measuring how long it took for the immobilized fluorescent beads to become liberated from the gel mesh and exhibit Brownian motion. To measure degradation by bacteria, a similar procedure was used but plasmin was replaced with bacteria. Glycerol stocks of bacterial strains (*Bacillus thuringiensis* Berliner ATCC #35646,

Bacillus subtilis subsp. *spizizenii* Nakamura et al. ATCC #6633, *Bacillus cereus* Frankland and Frankland ATCC #10987, *Bacillus circulans* Jordan ATCC #4513, *Staphylococcus epidermidis* (Winslow and Winslow) Evans ATCC #14990, *Staphylococcus aureus* subsp. *aureus* Rosenbach ATCC #25923) were streaked onto Difco™ LB agar, Lennox (BD Biosciences, San Jose, CA) plates. The bacteria were cultured overnight at 37°C. Colonies were picked and resuspended in the gel reaction buffer at 0.1 mg/mL. The suspended bacteria (2.78 μL) were then added to 5 μL pre-formed BNC-material (final bacteria concentration 36 μg/mL) and degradation was measured.

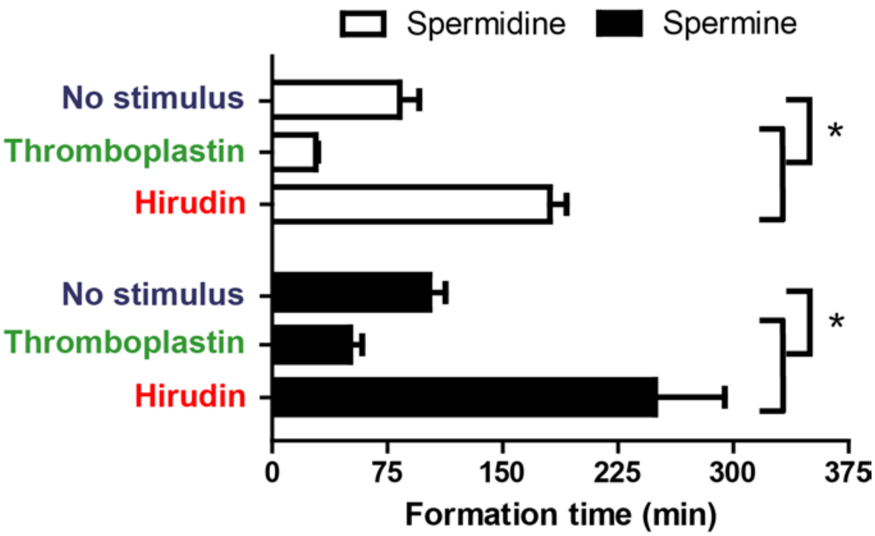

Supplementary Figure S1. Two different polyamines can be used to form the BNC-material. With either spermidine (which was used in the figures in the main text) or spermine, the BNC-material gelled faster or slower than inert controls in response to a known coagulation activator, thromboplastin, or an inhibitor, hirudin, respectively. $^{*}p < 0.05$. Data indicate mean ± SEM. $n = 3\text{-}5$.

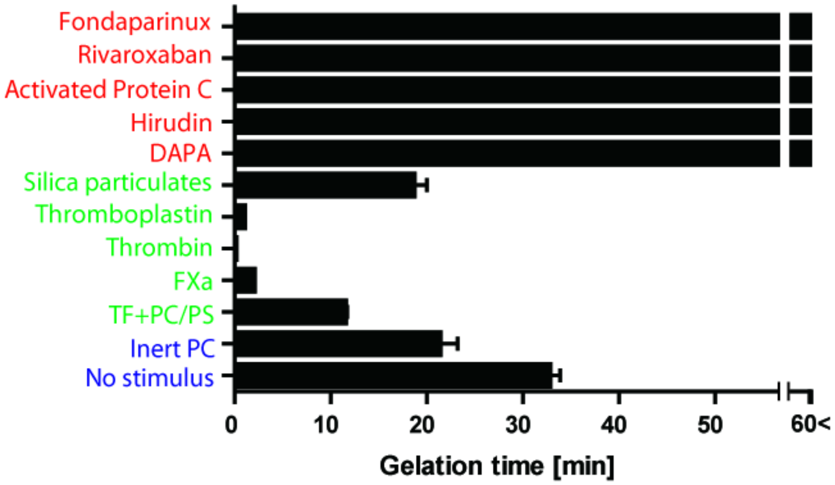

Supplementary Figure S2. The formation time of fibrin responds to diverse stimuli. Fibrin clots generated in recalcified normal plasma gelled faster or slower compared to the inert controls (blue) in response to known coagulation activators (green) and inhibitors (red), respectively. Data indicate mean ± SEM. *n* = 3.

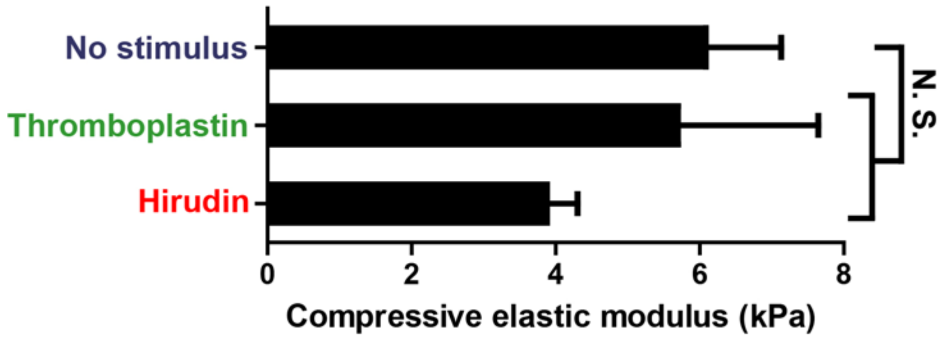

Supplementary Figure S3. Although modulators influence formation time, the physical properties of the final BNC-material are similar. The compressive elastic modulus of BNC-material gels formed in the presence of a known coagulation activator, thromboplastin, and an inhibitor, hirudin, were not significantly different from inert controls (all $p > 0.05$). Data indicate mean ± SEM. *n* = 3-4.

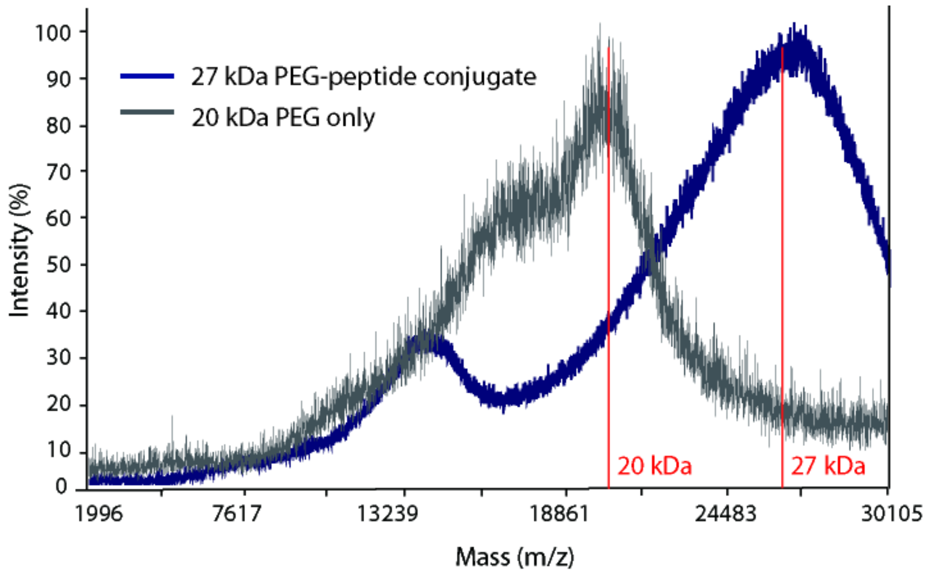

Supplementary Figure S4. The molecular weights of PEG-mal and the resulting PEG-peptide conjugate, measured by MALDI-TOF. To facilitate this mass spec analysis, 20 kDa, 4-armed PEG-mal was used rather than 40 kDa, 8-armed PEG-mal. The 20 kDa, 4-armed PEG-mal was conjugated with four 1.7 kDa peptides.

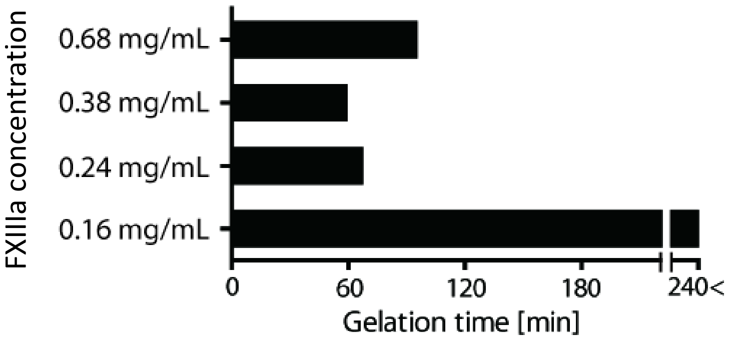

Supplementary Figure S5. Gelation times of the BNC-material displays a threshold response to the FXIIIa concentration. Undesired precipitation occurred at 0.68 mg/ml of FXIIIa.

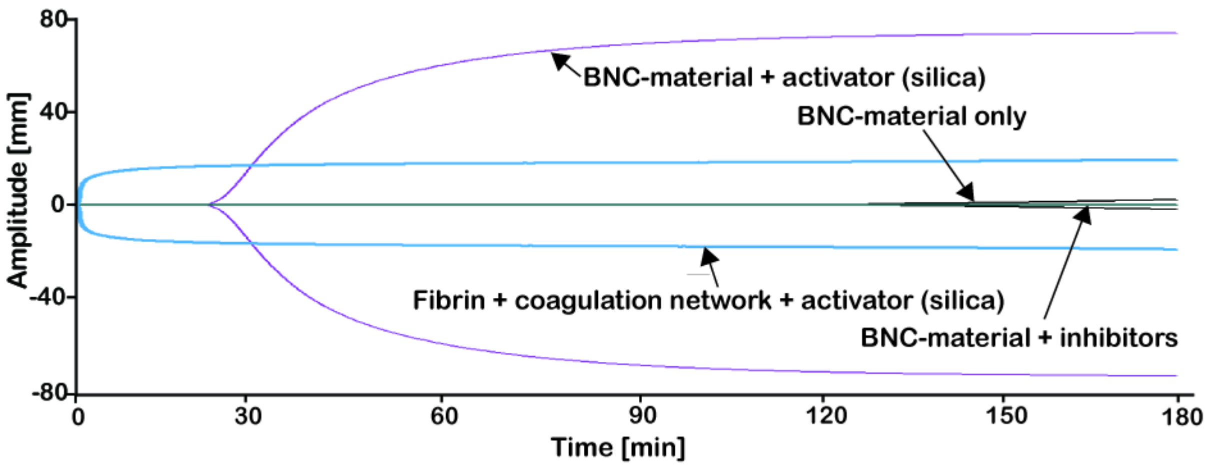

	Reaction time (R, min)	Maximum amplitude (MA, mm)	Elastic modulus (G, X100 N/m^2)
**BNC-material + activator (silica)**	17.8	72.5	13.2
**BNC-material only**	130.8	5.9	0.3
**Fibrin + coagulation network + activator (silica)**	0.8	17.0	1.0
**BNC-material + inhibitor of FXIIIa (T101)**	179.9	0	0
**BNC-material + inhibitors of proteases**	157	7.2	0.4

Supplementary Figure S6. The physical properties of the BNC-material change when coagulation is activated. The shear elastic modulus of the activated BNC-material (purple

curve) was greater than the unstimulated BNC-material (black curve), normal plasma with fibrin (blue curve) and the BNC-material containing protease inhibitors (green curve) after 3 hr.

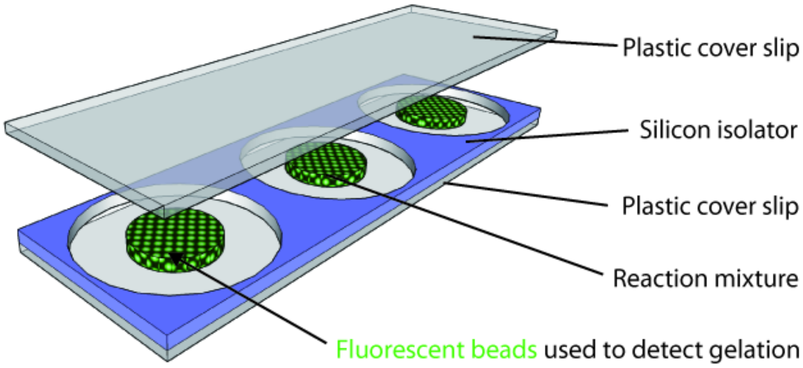

Supplementary Figure S7. Schematic of microchambers. Adhesive silicone isolators formed an air-tight seal once sandwiched between two coverslips. Each microchamber held a 5 μL sample.

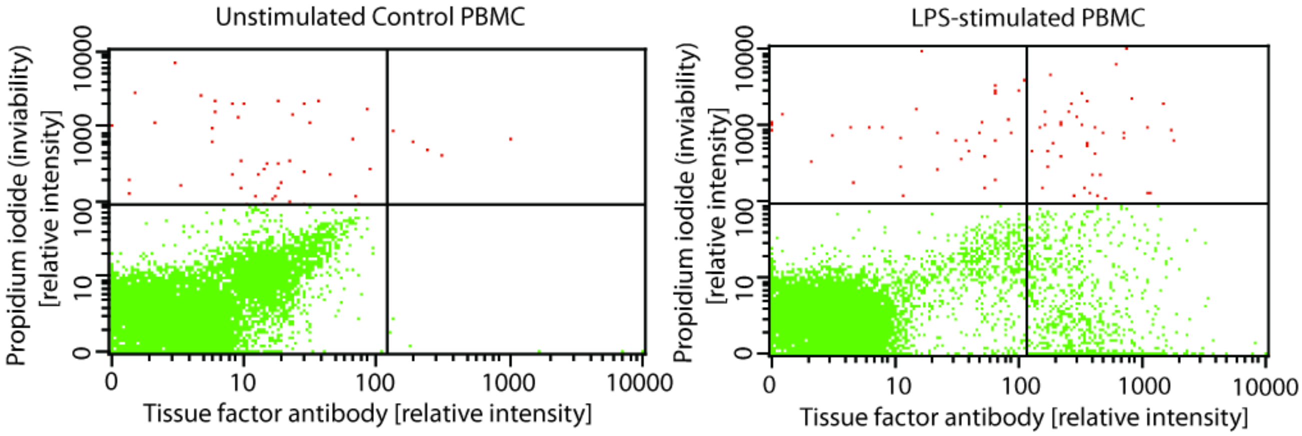

Supplementary Figure S8. PBMC were activated by LPS to express TF. The bottom right quadrant of both graphs shows target cells that are viable and express tissue factor. Viable TF-expressing cells were not consistently observed for the unstimulated control (0.06% of the population, shown on the plot on the left). A subpopulation of the PBMC expressed TF upon stimulation by LPS (6.98% of the population, shown on the plot on the right).

Supplementary Table S1. Enzyme activities of various plasma types. Three different types of frozen human plasma were obtained (Affinity Biologicals, Ancaster, Canada). Enzyme activity assays were performed by the manufacturer to determine activities.

Plasma Type	FII Activity	FV Activity	FVIII Activity	Fibrinogen Concentration
Normal plasma	1.12 U/ml	1.13 U/ml	1.22 U/ml	3.06 g/L
Fibrinogen-deficient plasma (active cascade)	1.02 U/mL	0.30 U/mL	0.64 U/mL	0.06 g/L
Fibrinogen-deficient and factors-depleted plasma *	0.79 U/mL	0.07 U/mL	0.29 U/mL	0.001 g/L

* FII, FV, and FVIII activities for this plasma are estimated based on the level of fibrinogen depletion relative to the other plasmas.

Supplementary Table S2. Formation times of the BNC-material with various concentrations of modulators.

Stimulus	Manufacturer	Final concentration	Polymer gelation time [min]
Human FXa	Haematologic Technologies Inc., Vermont, USA	**100 µg/mL**	33
		10 µg/mL	35
Bovine Thrombin	Sigma-Aldrich, St. Louis, USA	**53 units/mL**	31
		5.3 units/mL	31
		0.53 units/mL	47
Thromboplastin	Pacific Hemostasis, Middletown, USA	**6.6 mg/mL**	47
		0.66 mg/mL	29
		0.066 mg/mL	33
Silica Particulates	“Kontact,” Pacific Hemostasis, Middletown, USA	**13.3% (v/v)**	33
		1.33%	29
		0.133%	37
APC	Haematologic Technologies Inc., Vermont, USA	**50 µg/mL**	152
		5 µg/mL	89
DAPA	Haematologic Technologies Inc., Vermont, USA	**100 µg/mL**	100
		10 µg/mL	120
Hirudin	Sigma-Aldrich, St. Louis, USA	**319 units/mL**	232
		31.9 units/mL	194
Rivaroxaban	Sigma-Aldrich, St. Louis, USA	133 µM	125
		**13.3 µM**	204
		0.133 µM	171

Fondaparinux	"Xarelto," Bayer HealthCare, Berlin, Germany	133 µM<br>**13.3 µM**<br>0.133 µM	171<br>214<br>191
Silica (**Fig. 2f**)	"SiMAG-Silanol," Chemicell GmbH, Berlin, Germany	**66 µg/mL**	49
Hydroxyl-coated silica	"SiMAG-Hydroxyl," Chemicell GmbH, Berlin, Germany	**66 µg/mL**	198
Ellagic acid	Sigma-Aldrich, St. Louis, USA	**6.6 µM**	118
Rutin trihydrate	Sigma-Aldrich, St. Louis, USA	**6.6 µM**	278

Bolded values are the concentrations used in the main text.

Supplementary Table S3. Degradation of the BNC-material is dependent on the concentration of plasmin.

Final plasmin concentration	Polymer degradation time [min]
2000 µg/mL	76
200 µg/mL	124
20 µg/mL	>900

References for Supplementary Information

1 Shankarraman, V., Davis-Gorman, G., Copeland, J. G., Caplan, M. R. & McDonagh, P. F. Standardized methods to quantify thrombogenicity of blood-contacting materials via thromboelastography. *Journal of Biomedical Materials Research Part B-Applied Biomaterials* **100B**, 230-238 (2012).

2 McMahon, R., Hahn, M., Pendleton, M. & Ellis, E. A Simple Preparation Method for Mesh Fibrin Hydrogel Composites for Conventional SEM. *Microscopy and Microanalysis* **16**, 1030-1031 (2010).

3 Holzinger, A., Wasteneys, G. O. & Lutz, C. Investigating cytoskeletal function in chloroplast protrusion formation in the arctic-alpine plant Oxyria digyna. *Plant Biology* **9**, 400-410 (2007).

4 Böyum, A. Isolation of leucocytes from human blood. Further observations. Methylcellulose, dextran, and ficoll as erythrocyte aggregating agents. *Scandinavian Journal of Clinical & Laboratory Investigation* **97**, 31-50 (1968).
